# Medicare and Medicaid Dual-Eligible Special Needs Plan Enrollment and Beneficiary-Reported Experiences With Care

**DOI:** 10.1001/jamahealthforum.2023.2957

**Published:** 2023-09-08

**Authors:** David J. Meyers, Kendra Offiaeli, Amal N. Trivedi, Eric T. Roberts

**Affiliations:** 1 Department of Health Services, Policy, and Practice, Brown University School of Public Health, Providence, Rhode Island; 2 Department of Health Policy and Management, University of Pittsburgh School of Public Health, Pittsburgh, Pennsylvania

## Abstract

This cross-sectional study analyzes Medicare Advantage surveys to compare Medicare and Medicaid dual-eligible individuals’ experiences with care across 3 established categories of plans.

## Introduction

People dually eligible for Medicare and Medicaid are among individuals with the greatest need in the US^1^ and have enrolled in Medicare Advantage (MA) plans at a pace faster than non–dual-eligible individuals.^2^ To improve care for this population, the Centers for Medicare & Medicaid Services (CMS) has encouraged development of dual-eligible special needs plans (D-SNPs)—MA plans that exclusively serve dual-eligible beneficiaries, coordinate Medicare and Medicaid benefits, and, in some cases, manage Medicaid spending.^3^

These D-SNPs vary in the extent to which they manage Medicaid spending. Coordination-only D-SNPs (60.6% of D-SNPs in 2023) provide limited care coordination (eg, notifying Medicaid when enrollees are hospitalized) but do not manage Medicaid spending. Fully integrated D-SNPs (FIDE-SNPs) (8.0% of D-SNPs) have capitation contracts to manage Medicaid long-term care and behavioral health care spending.^4,5^ Limited evidence exists on whether greater integration of Medicare and Medicaid spending is associated with improved quality of care.^4,5^

We analyzed MA Consumer Assessment of Healthcare Providers and Systems (CAHPS) surveys to compare dual-eligible individuals’ experiences with care across 3 categories of plans: coordination-only D-SNPs, FIDE-SNPs, and non–D-SNP MA plans.

## Methods

We analyzed respondent-level MA-CAHPS data from 2015-2018 for dual-eligible individuals with full Medicaid (mean response rate, 43%). MA-CAHPS assesses respondent ratings of health plans and patient experiences with care in domains such as coordination. This cross-sectional study met the STROBE reporting guideline and was deemed exempt with a waiver of informed consent from the Brown University Institutional Review Board for its use of secondary data.

We used administrative data linked to MA-CAHPS respondents to identify enrollees in coordination-only D-SNPs, FIDE-SNPs, and non–D-SNP MA plans in the survey month. Respondents with more than 100 days of nursing home care in the survey year and in institutional and chronic condition SNPs were excluded.

Outcomes were 6 composite and 3 single-item measures from MA-CAHPS following CMS protocol for performance measurement (eMethods in Supplement 1). We estimated a respondent-level linear regression for each outcome as a function of plan category, adjusting for respondent demographics, original Medicare entitlement, physical and mental health status, state fixed effects, and use of a proxy respondent. Models were estimated with plan random effects to account for clustering by plan. Data were analyzed from January to March 2023. Statistical significance for adjusted mean differences was defined as a 95% CI excluding zero.

## Results

Our sample included 181 806 MA-CAHPS respondents, of whom 30.05% were in coordination-only D-SNPs; 5.81%, in FIDE-SNPs; and 64.14%, in non–D-SNP MA plans. FIDE-SNP respondents were less often disabled and more likely to use a proxy respondent than coordination-only D-SNP and non–D-SNP respondents (Table).

**Table.** Characteristics of the Sample by Medicare Advantage Plan Type

Characteristic	Medicare Advantage plan type^a^		
	Non–D-SNP Medicare Advantage plans^b^^,^^c^	FIDE-SNPs^c^	Coordination-only D-SNPs^c^
No. (row %)	116 616 (64.14)	10 565 (5.81)	54 625 (30.05)
Female^d^	75 496 (64.74)	7096 (67.17)	35 136 (64.32)
Male^d^	41 120 (35.26)	3469 (32.83)	19 489 (35.68)
Ethnicity^d^			
Hispanic or Latino	23 570 (21.61)	2560 (25.74)	12 859 (25.34)
Non-Hispanic or Latino	85 507 (78.39)	7387 (74.26)	37 877 (74.66)
Race^d^			
American Indian or Alaska native	511 (0.46)	42 (0.40)	389 (0.71)
Asian	23 554 (21.10)	2583 (24.45)	12 889 (23.60)
Black	27 233 (24.40)	1697 (16.06)	13 978 (25.59)
Hispanic or Latino	6238 (5.59)	786 (7.44)	3617 (6.62)
White	57 427 (51.45)	5202 (49.24)	22 850 (41.83)
Other	887 (0.79)	106 (1.00)	483 (0.88)
Unknown	766 (0.69)	149 (1.41)	419 (0.77)
Age, mean (SD), y^e^	68.4 (13.0)	73.9 (11.5)	65.9 (13.2)
Educational level^d^			
Eighth grade or less	20 334 (18.22)	2582 (26.00)	10 332 (20.15)
Some high school	20 484 (18.35)	1693 (17.05)	10 105 (19.71)
High school graduate/GED	36 859 (33.03)	3085 (31.07)	17 404 (33.95)
Some college or 2-y degree	23 229 (20.81)	1609 (16.20)	9985 (19.47)
4-y College graduate	5347 (4.79)	537 (5.41)	2056 (4.01)
More than 4-y college degree	5347 (4.79)	424 (4.27)	1389 (2.71)
Original reason for Medicare entitlement^e^			
Old age and survivor’s insurance	62 067 (53.22)	6997 (66.23)	25 491 (46.67)
Disability insurance benefits	54 549 (46.78)	3568 (33.77)	29 134 (53.33)
Proxy respondent^d^	24 151 (21.39)	3407 (33.22)	11 188 (21.24)
Overall general health self-rating^d^			
Excellent	6169 (5.42)	544 (5.26)	3014 (5.64)
Very good	16 139 (14.18)	1330 (12.87)	6948 (13.01)
Good	38 218 (33.59)	3357 (32.49)	17 167 (32.14)
Fair	40 597 (35.68)	3884 (37.59)	20 129 (37.68)
Poor	12 659 (11.13)	1218 (11.79)	6162 (11.54)
Overall mental health self-rating^d^			
Excellent	16 376 (14.38)	1325 (12.81)	7301 (13.66)
Very good	24 401 (21.42)	2058 (19.90)	10 055 (18.81)
Good	37 788 (33.17)	3465 (33.50)	17 769 (33.24)
Fair	28 263 (24.81)	2756 (26.65)	14 705 (27.51)
Poor	7089 (6.22)	739 (7.14)	3627 (6.78)
Unadjusted measures, mean (SD)^d^^,^^f^			
Getting needed care	80.7 (25.3)	81.7 (22.7)	80.7 (24.3)
Getting appointments and care quickly	74.1 (21.3)	77.1 (19.8)	74.3 (21.3)
Physicians who communicate well	88.8 (18.7)	88.9 (17.8)	88.3 (19.4)
Customer service	82.1 (19.6)	82.9 (18.5)	83.3 (18.9)
Getting needed prescription drugs	77.7 (16.5)	76.9 (14.7)	76.5 (15.5)
Care coordination	89.7 (17.7)	88.2 (18.4)	88.0 (18.6)
Rating of health plan	84.9 (20.6)	89.0 (17.5)	86.6 (19.9)
Rating of health plan quality	82.3 (21.9)	83.7 (20.4)	82.0 (22.1)
Rating of drug plan	87.1 (19.3)	89.9 (16.9)	87.8 (18.9)

Abbreviations: D-SNP, dual-eligible special needs plan; FIDE-SNP, fully integrated D-SNP; GED, general equivalency diploma.

^a^ Data are given as number (percentage) of total unless otherwise indicated. Dual-eligible individuals receiving more than 100 days of institutional care in the survey year and those enrolled in institutional or chronic condition special needs plans were excluded.

^b^ Included full-benefit dual-eligible individuals were enrolled in a Medicare Advantage plan, not an FIDE-SNP or coordination-only D-SNP.

^c^ Plan enrollment assessed from the Medicare Beneficiary Summary File in the survey month.

^d^ Variable is self-reported by respondents to the Medicare Advantage Consumer Assessment of Healthcare Providers and Systems survey. The unknown category for race and ethnicity was self-reported and represents when race and ethnicity were otherwise missing from the self-report.

^e^ Assessed from the Medicare Beneficiary Summary File in the survey year.

^f^ The first 6 of these measures are calculated as composites, and the last 3 are single-item questions. All these measures are scaled from 0 to 100, with 100 representing the highest possible score on the measure.

Compared with non–D-SNP MA plans, respondents in FIDE-SNP plans reported significantly lower ratings of care coordination (adjusted mean difference: −1.8; 95% CI, −2.3 to −1.4) and getting needed prescription drugs (−1.9; 95% CI, −3.4 to −0.3) but higher ratings of their plan (2.5; 95% CI, 1.9 to 3.1), prescription drug coverage (1.3; 95% CI, 0.79 to 1.8), getting appointments and care quickly (1.1; 95% CI, 0.0 to 2.2), customer service (1.5; 95% CI, 0.3 to 2.7), and care quality rating (0.8; 95% CI, 0.2 to 1.4) (Figure).

**Figure.**
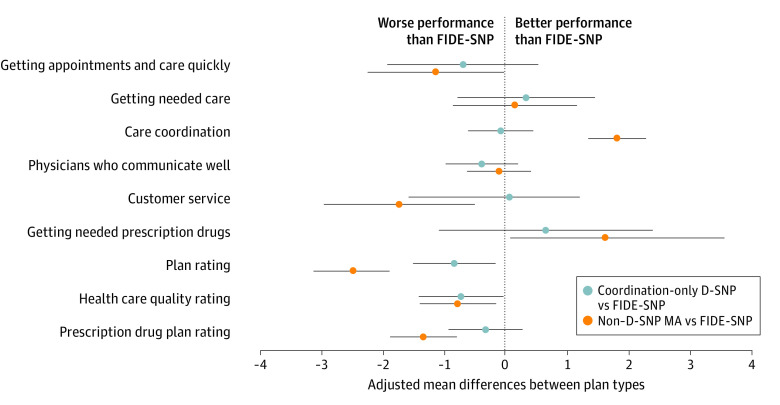
Adjusted Differences in Outcomes by Type of Dual-Eligible Special Needs Plan (D-SNP) or Non–D-SNP Medicare Advantage (MA) Plan The reference group at 0 in the figure is fully integrated D-SNP (FIDE-SNP). Each difference represents an adjusted mean difference from our primary regression models. Scores in each domain are calculated from responses to the MA Consumer Assessment of Healthcare Providers and Systems survey following Centers for Medicare & Medicaid Services methods and were scaled from 0 to 100. All estimates are from linear regression models that adjusted for age, sex, race, ethnicity, educational level, proxy status, original reason for Medicare entitlement, overall physical health status, overall mental health status, state fixed effects, and plan random effects. The 95% CIs are clustered by plan.

Respondents in FIDE-SNPs reported significantly higher ratings than those in coordination-only D-SNPs for plan rating (0.8; 95% CI, 0.2 to 1.5) and health care quality rating (0.7; 95% CI, 0.0 to 1.4). Differences between FIDE-SNPs and coordination-only D-SNPs in other outcomes were not statistically significant.

## Discussion

In this cross-sectional study, we found that FIDE-SNPs performed better than non–D-SNP MA plans in some domains but worse on domains including care coordination. Furthermore, FIDE-SNPs generally did not perform better than coordination-only D-SNP plans. The findings highlight some benefits in patient experience associated with enrollment in FIDE-SNPs and an opportunity to improve patient experience in these plans. This is particularly salient in areas such as care coordination, where integration could be improved. Study limitations included potential confounding from unmeasured enrollee differences across plans and analysis of the years preceding stronger federal regulations governing D-SNPs.^3,6^ As D-SNPs evolve, continued monitoring is needed to ensure these plans provide increased value for dual-eligible individuals.

## Notes

Supplement 1.
eMethods.

Supplement 2.
Data Sharing Statement

## References

[1] Medicaid and CHIP Payment and Access Commission. Data book: beneficiaries dually eligible for Medicare and Medicaid. 2018. Accessed June 2023. https://www.macpac.gov/publication/data-book-beneficiaries-dually-eligible-for-medicare-and-medicaid-3/

[2] Meyers DJ, Mor V, Rahman M, Trivedi AN. Growth in Medicare Advantage greatest among Black and Hispanic enrollees. Health Aff (Millwood). 2021;40(6):945-950. doi:10.1377/hlthaff.2021.00118 34097525 PMC8297509

[3] Medicaid and CHIP Payment and Access Commission. Chapter 4: establishing a unified program for dually eligible beneficiaries: design considerations. March 2021. Accessed June 2023. https://www.macpac.gov/publication/march-2021-report-to-congress-on-medicaid-and-chip/

[4] Haviland AM, Elliott MN, Klein DJ, Orr N, Hambarsoomian K, Zaslavsky AM. Do dual eligible beneficiaries experience better health care in special needs plans? Health Serv Res. 2021;56(3):517-527. doi:10.1111/1475-6773.13620 33442869 PMC8143688

[5] Roberts ET, Mellor JM. Differences in care between special needs plans and other Medicare coverage for dual eligibles. Health Aff (Millwood). 2022;41(9):1238-1247. doi:10.1377/hlthaff.2022.00463 36067441 PMC9575653

[6] Centers for Medicare & Medicaid Services. Additional guidance on CY 2021 Medicare-Medicaid integration requirements for dual eligible special needs plans (D-SNPs). 2020. Accessed June 2023. https://www.cms.gov/files/document/cy2021dsnpsmedicaremedicaidintegrationrequirements.pdf

