# Supplementary material for: Medicare and Medicaid Dual-Eligible Special Needs Plan Enrollment and Beneficiary-Reported Experiences With Care
Source: JAMA Health Forum. 2023 Sep 8;4(9):e232957. doi: 10.1001/jamahealthforum.2023.2957 (PMC10492180; doi:10.1001/jamahealthforum.2023.2957)
Supplement: Supplement 1. — eMethods. [file jamahealthforum-e232957-s001.pdf]

Supplementary Online Content

Meyers DJ, Offiaeli K, Trivedi AN, Roberts ET. Medicare and Medicaid dual-eligible special needs plan enrollment and beneficiary-reported experiences with care. *JAMA Health Forum*. 2023;4(9):e232957. doi:10.1001/jamahealthforum.2023.2957

eMethods.

This supplementary material has been provided by the authors to give readers additional information about their work.

eMethods.

MA & PDP CAHPS Survey

Our primary source of data was the MA & PDP Consumer Assessment of Healthcare Providers and Systems (MA-CAHPS) from 2015-2018. The CAHPS survey is a fielded to a sample of around 800 beneficiaries in each MA contract each year and has an average response rate of 45% during our study years. The survey is primarily conducted over mail, but telephone follow up is used for non-response. The CAHPS surveys used for this study are availible as Researcher Identifiable Files (RIFs). The data are released at the individual level, and linkable to other sources of CMS data using a beneficiary ID. We used that linkage to identify if a beneficiary was eligible for our study by being dually eligible, and if they were enrolled in a Special Needs Plan or other Medicare Advantage plan.

Outcome Measures

For our primary outcomes we used 6 composite measures calculated from the CAHPS surveys and three stand alone measures. The composite measures, listed below, are calculated by CMS and used for performance measurement of MA contracts and are incorporated into contract star ratings. Each composite combines several questions together and is then scaled to 0-100. The single measures are asked on a scale of 1-10 and then multiplied by 10 so that they are on the same scale as the composite measures. The composite measures are listed below:

Getting Needed Care:

In the last 6 months, how often was it easy to get the care, tests or treatment you needed? (never, sometimes, usually, always)

In the last 6 months, how often did you get an appointment to see a specialist as soon as you needed? (never, sometimes, usually, always)

Getting Care Quickly:

In the last 6 months, when you needed care right away, how often did you get care as soon as you needed? (never, sometimes, usually, always)

In the last 6 months, how often did you get an appointment for a check-up or routine care as soon as you needed? (never, sometimes, usually, always)

Doctors Who Communicate Well

In the last 6 months, how often did your personal doctor explain things in a way that was easy to understand? (never, sometimes, usually, always)

In the last 6 months, how often did your personal doctor listen carefully to you? (never, sometimes, usually, always)

In the last 6 months, how often did your personal doctor show respect for what you had to say? (never, sometimes, usually, always)

© 2023 Meyers DJ et al. *JAMA Health Forum.*

In the last 6 months, how often did your personal doctor spend enough time with you? (never, sometimes, usually, always)

Customer Service

In the last 6 months, how often did your health plan's customer service give you the information or help you needed? (never, sometimes, usually, always)

In the last 6 months, how often did your health plan's customer service staff treat you with courtesy and respect? (never, sometimes, usually, always)

In the last 6 months, how often were the forms from your health plan easy to fill out? (never, sometimes, usually, always)

Getting Needed Prescription Drugs

In the last 6 months, how often was it easy to use your prescription drug plan to get the medicines your doctor prescribed? (never, sometimes, usually, always)

In the last 6 months, how often was it easy to use your prescription drug plan to fill a prescription at your local pharmacy? (never, sometimes, usually, always)

In the last 6 months, how often was it easy to use your prescription drug plan to fill a prescription by mail? (never, sometimes, usually, always)

Care Coordination

In the last 6 months, when you visited your personal doctor for a scheduled appointment, how often did he or she have your medical records or other information about your care? (never, sometimes, usually, always)

In the last 6 months, when your personal doctor ordered a blood test, x-ray or other test for you, how often did someone from your personal doctor's office follow up to give you those results? (never, sometimes, usually, always)

In the last 6 months, when your personal doctor ordered a blood test, x-ray or other test for you, how often did you get those results as soon as you needed them? (never, sometimes, usually, always)

In the last 6 months, how often did you and your personal doctor talk about all the prescription medicines you were taking? (never, sometimes, usually, always)

In the last 6 months, did you get the help you needed from your personal doctor's office to manage your care among these different providers and services? (never, sometimes, usually, always)

In the last 6 months, how often did your personal doctor seem informed and up-to- date about the care you got from specialists? (never, sometimes, usually, always)

© 2023 Meyers DJ et al. *JAMA Health Forum.*
