# Supplementary material for: Medicare and Medicaid Dual-Eligible Special Needs Plan Enrollment and Beneficiary-Reported Experiences With Care
Source: JAMA Health Forum. 2023 Sep 8;4(9):e232957. doi: 10.1001/jamahealthforum.2023.2957 (PMC10492180; doi:10.1001/jamahealthforum.2023.2957)
Supplement: Supplement 2. — Data Sharing Statement [file jamahealthforum-e232957-s002.pdf]

Data Sharing Statement

Meyers. Medicare and Medicaid Dual-Eligible Special Needs Plan Enrollment and Beneficiary-Reported Experiences With Care. *JAMA Health Forum*. Published September 08, 2023. doi:10.1001/jamahealthforum.2023.2957

Data
Data available: No

Additional Information
Explanation for why data not available: The data for this study is only available under a Data Use Agreement with the Centers for Medicare and Medicaid Services.
